# Optimizing functional imaging protocols for assessing the outcome of fetal cell transplantation in Parkinson's disease

**DOI:** 10.1186/1741-7015-9-50

**Published:** 2011-05-10

**Authors:** Marios Politis

**Affiliations:** 1 Centre for Neuroscience, Department of Medicine, Hammersmith Hospital, Imperial College London, London W12 0NN, UK

## Abstract

Clinical trials aiming to assess the safety and efficacy of fetal cell transplantation in Parkinson's disease rely on the hypothesis that the grafted tissue will survive and grow, restore striatal dopaminergic neurotransmission, improve the connectivity between striatum, thalamus and cortex and, thereby, produce long-lasting clinical improvement while avoiding the development of adverse effects. Although transplantation of human fetal ventral mesencephalic tissue has been reported as one of the most effective reparative therapies in Parkinson's disease patients to date, different studies have shown inconsistent results causing a paucity of new trials over the last decade. However, during this period, functional imaging alongside other scientific developments from clinical observations and animal work has significantly aided in understanding the mechanisms responsible for the success or failure of grafting human fetal tissue. Recent advances in functional imaging including both positron emission tomography and functional magnetic resonance imaging could be proven useful *in vivo *tools for the development and assessment of new clinically competitive trials. In this commentary we discuss how an optimized functional imaging protocol could assist new clinical trials using fetal cell transplantation in Parkinson's disease.

## Introduction

In previous trials, functional imaging using mainly positron emission tomography (PET) has provided objective *in vivo *evidence that human dopamine (DA) -rich fetal ventral mesencephalic (VM) tissue implanted in the striatum of Parkinson's disease (PD) patients can survive, grow, release DA, normalize brain metabolism and restore striatal-cortical connections, clinically corresponding to significant symptomatic relief in some cases [1-3].

PET with ^18^F-DOPA (see Table 1 for explanation of PET ligands) has been consistently used since the early open-label [4-8] up to the more recent double-blind sham-surgery controlled [9,10] clinical trials to objectively monitor survival and growth of human fetal DA neurons grafted in the striatum of PD patients. PET imaging using ^11^C-raclopride after placebo or methamphetamine administration has shown that graft-derived DA cells were able to restore the release of endogenous DA in the striatum of PD patients to almost normal levels [11]. Longitudinal assessments with H_2_^15^O PET following transplantation have shown a gradual restoration of movement-related activation in motor cortical areas indicating that grafted cells can form connections and are able to restore striato-cortical networks in the host brain [12].

**Table 1:** Positron emission tomography techniques

Technique	Target	Use
^18^F-DOPA PET	Aromatic amino acid decarboxylase (AADC)	Provides measures of AADC activity and allows an indirect measure of DA storage within the DA terminals.
^11^C-raclopride PET	Post-synaptic DA D2 receptors	Is a subject to competitive displacement by endogenous DA. Acute administration of substances such as amphetamine, methylphenidate, or L-DOPA, which are known to increase the levels of extracellular DA result in a reduction of ^11^C-raclopride binding.
H_2_^15^O PET	Regional cerebral blood flow (rCBF)	Brain activation and regional cerebral metabolism.
^76^Br-FE-CBT PET	Dopamine transporter (DAT)	Marker of presynaptic DA terminals integrity and DAT availability
^123^I-IPT SPECT	DAT	Marker of presynaptic DA terminals integrity and DAT availability
^123^I-FP-CIT SPECT	DAT	Marker of presynaptic DA terminals integrity and DAT availability
^11^C-PK11195 PET	Translocator protein (TSPO)	Is an 18 kDa protein of the outer mitochondrial membrane that is upregulated with activation of microglia
^11^C-DASB PET	5-HT transporter (SERT)	Marker of presynaptic 5-HT terminals integrity and SERT availability
^11^C-dihydrotetrabenazine (DTBZ) PET	Vesicular monoamine transporter (VMAT2)	Marker of presynaptic monoaminergic terminals integrity

Advances in imaging techniques including both PET and magnetic resonance imaging (MRI) could further assist in the development and monitoring of new clinically competitive trials.

## Discussion

Despite the lack of new clinical trials since the beginning of the previous decade, retrospective analysis of data, studies in animal models and long-term clinical and imaging observations in the earlier transplanted PD patients have allowed us to understand a number of ways that functional imaging could help in understanding and monitoring outcomes of fetal cell transplantation trials in PD.

There are now up to 16 years of post-transplantation ^18^F-DOPA PET follow-up data showing graft viability and continuous clinical benefit in several transplanted PD patients with fetal VM tissue [11,13-15]. As the degree of motor impairment has been shown to correlate with ^18^F-DOPA uptake in the striata of PD patients [16], clinical outcomes following fetal cell transplantation could be associated with the number of viable DA cells innervating the striatum as assessed by ^18^F-DOPA PET. This notion could explain the differences in the outcomes between open-label trials reporting an average of 50 to 85% increase in ^18^F-DOPA uptake associated with good clinical improvement of PD motor symptoms and reductions in medication requirements [4-8], and the two double-blind sham-surgery controlled clinical trials reporting an average of 20 to 40% increases in ^18^F-DOPA uptake associated with poor clinical outcomes during the first periods post-transplantation [9,10].

^18^F-DOPA PET can also help facilitate patient selection and screening as patients with baseline reductions in ^18^F-DOPA uptake extending to the ventral part of the striatum and patients with reductions in ^18^F-DOPA uptake consistent with atypical or secondary Parkinsonism should be excluded from these trials [13,17]. This knowledge, which derived from *post-hoc *analysis, raises the possibility of a time window for optimal transplantation outcomes as preoperative preservation of DA innervation in ventral striatal areas seems to be predictive of a better outcome.

During past years, a small number of studies have been undertaken in order to demonstrate imaging of DA transporters (DAT) in the grafted striatum. Imaging of DAT with PET or single photon emission computed tomography (SPECT) could be used as an alternative technique to ^18^F-DOPA PET. However, in line with the inconsistent results observed in *post-mortem *studies with regard to DAT expression in the grafted neurons [18,19], ^76^Br-FE-CBT PET failed to visualize DAT in grafted striata enhancing ^18^F-DOPA [20], whereas ^123^I-IPT [21] and ^123^I-FP-CIT [15] SPECT showed robust DAT availability and compatible uptake to ^18^F-DOPA.

Up to 10 years post-transplantation follow-up data using ^11^C-raclopride PET have shown several cases where graft-derived DA cells were able to restore the release of endogenous DA in the striatum of PD patients [11,14,17]. The results of these studies suggested that it is very likely that the efficient restoration of DA release in large parts of the grafted striatum underlies patients' clinical improvement of motor symptoms. Moreover, from PET studies using H_2_^15^O we learned that despite an early stabilization of DA reinnervation in the striatum, graft function and integration continue and symptomatic relief may also require the functional reafferentation of striato-thalamo-cortical circuitries in the host brain [12].

Immunosuppression could be another factor related to the outcomes of clinical trials, although the role of neuroinflammation in influencing the outcome of transplantation procedures in PD is not known. There is evidence showing that long-term immunosuppresion can be withdrawn without interfering with graft survival or the motor recovery induced by transplantation [17]. Additional evidence indicated that the occurrence of graft-induced dyskinesias (GIDs), one of the most disabling side effects of cell therapy in PD, could be induced by inflammatory and immune responses around the graft. In one study GIDs developed after discontinuation of immunosuppressive therapy, with signs of an inflammatory reaction around the grafts in autopsied cases [10]. Although imaging studies have never been employed, PET imaging can be used to explore the possible role of host inflammatory reaction in relation to fetal cell transplantation outcomes in PD using markers of microglial activation such as ^11^C-PK11195 PET or any of the other recently developed translocator protein (TSPO) PET ligands.

GIDs are a severe adverse effect of fetal cell transplantation in PD hindering the further development of cell transplantation trials [9,10,14,15,22-25]. Notwithstanding the several theories proposed [25], recent data imply that the development of GIDs could be related to the composition of grafted tissue [14], as human fetal VM tissue contains a varied proportion of non-DA neurons [26], including serotonin (5-HT) neurons. ^11^C-DASB PET and a clinical trial with an agent dampening transmitter release from 5-HT neurons was used to show a causal relationship between mishandling of DA release from the graft-derived 5-HT hyperinnervation in the striatum of PD patients and the occurrence of GIDs [14,15]. Furthermore, it was suggested that the high ratios between 5-HT and DA neurons and between 5-HT transporters (SERT) and DAT could be the driving factor for the development of GIDs [14,15,25]. Therefore, these results suggest that achieving normal striatal 5-HT/DA and SERT/DAT ratios following transplantation of fetal tissue or stem cells should be necessary to avoid the development of GIDs.

Recent advances in MRI techniques allow us to examine the brain in a way that was not previously possible. The examination of functional connectivity between brain regions is now possible using resting-state functional MRI (fMRI). These resting state networks (RSNs) are believed to reflect functional communication between brain regions and could be very informative in providing insights of large-scale neuronal communication during restorative therapies such as fetal cell transplantation in PD [27-29]. FMRI paradigms with motor execution (ME) tasks are now widely employed to understand brain activation in the corresponding regions. Diffusion Tensor Imaging (DTI) offers a method to assess white matter (WM) structural connectivity and thereby, alterations underlying neurodegenerative diseases such as PD and the potential effects of restorative therapies [28,30].

## Conclusions

A new European Commission-funded multicenter trial under the name Transeuro was recently launched for a new round of fetal cell transplantation trials http://www.transeuro.org.uk. Functional imaging protocols for this or any other future trials employing fetal or stem cells will undoubtedly benefit from the lessons learned from over two decades of research.

An optimized functional imaging protocol (Table 2) using PET should search for a ligand tagging a specific to DA presynaptic terminal location in order to assess the DA-rich graft survival and growth. A good candidate is DAT; however, post-mortem and *in vivo *imaging results with PET and SPECT have been inconsistent. ^11^C-dihydrotetrabenazine (DTBZ) PET measuring the density of the vesicular monoamine transporter (VMAT2) could serve as an alternative, but to date there are no ^11^C-DTBZ PET data on the expression and survival of VMAT2 in VM tissue grafts and VMAT2 is also present in other monoaminergic systems. Therefore, ^18^F-DOPA PET remains today the standard for monitoring survival and growth of grafted DA cells. According to data from open-label and double blind trials it appears that a short-term increase in ^18^F-DOPA uptake of more than 50% from baseline is necessary to achieve clinically valuable antiparkinsonian effects. Measures of ^18^F-DOPA uptake at baseline can also provide valuable information to assist in the selection of patients for trials of cell-based DA therapies in PD by excluding those showing reduced uptake in the ventral striatum. ^11^C-raclopride PET together with a competitive displacement challenge (for example, amphetamine, methylphenidate, or L-DOPA) could be used to visualize a graft's ability to release DA. ^11^C-DASB PET together with ^18^F-DOPA PET and markers of DAT availability can be used to calculate binding ratios reflecting proportions of 5-HT to DA neurons and SERT to DAT binding sites and, therefore, assist in the preparation of grafts during preoperational screening and, hence, prevent and monitor the development of GIDs postoperational. Inflammatory and immune responses around the graft alongside the effect of immunosuppressive therapy on transplantation outcomes could be assessed with ^11^C-PK11195 or one of the other recently developed TSPO PET ligands. Moreover, the new MRI techniques such as RSNs fMRI and DTI could assist in the assessment of functional and WM structural connectivity between the graft and the host brain while ME tasks could be employed replacing previous paradigms with H_2_^15^O PET.

**Table 2:** Examples of imaging techniques used in the past and that can be used in the future

Assessments	Previous functional imaging protocols	Optimized functional imaging protocols
DA-rich graft survival and growth	^18^F-DOPA PET	^18^F-DOPA PET
Patient selection	-	^18^F-DOPA PET
DA release from the graft	^11^C-raclopride with challenge	^11^C-raclopride with challenge
Graft-derived 5-HT innervation and growth	-	^11^C-DASB
Graft-induced dyskinesias	-	^11^C-DASB PET/^18^F-DOPA PET ratio ^11^C-DASB PET/^123^I-FP-CIT SPECT ratio
Inflammatory and immune responses around the graft	-	^11^C-PK11195
Brain activation during movement	H_2_^15^O PET with motor execution tasks	fMRI with motor execution tasks
Functional connectivity between brain regions	-	Resting-state fMRI
White matter structural connectivity	-	Diffusion Tensor Imaging

Although functional imaging cannot currently be used as a primary endpoint in clinical transplantation trials, if used appropriately, it can provide researchers with an additional valuable *in vivo *tool alongside clinical observations. It is worth noting, however, that previous knowledge has shown that in order to efficiently monitor cell replacement therapies in PD with functional imaging, long period follow-up assessments are needed and conclusions cannot be ultimately achieved with short follow-up periods.

## Abbreviations

5-HT: serotonin; ^11^C-DTBZ: ^11^C-dihydrotetrabenazine; DA: dopamine; DAT: dopamine transporter; DTI: Diffusion Tensor Imaging; fMRI: functional magnetic resonance imaging; GIDs: graft-induced dyskinesias; ME: motor execution; PD: Parkinson's disease; PET: positron emission tomography; RSNs: resting state networks; SERT: serotonin transporter; SPECT: single photon emission computed tomography; TSPO: translocator protein; VM: ventral mesencephalic; VMAT2: vesicular monoamine transporter 2; WM: white matter.

## Competing interests

The author declares that they have no competing interests.

## Authors' contributions

MP is entirely responsible for the content of this article.

## Pre-publication history

The pre-publication history for this paper can be accessed here:

http://www.biomedcentral.com/1741-7015/9/50/prepub
